# Initiation rates of statin therapy for the primary prevention of cardiovascular disease: an assessment of differences between countries of the UK and between regions within England

**DOI:** 10.1136/bmjopen-2014-007207

**Published:** 2015-03-06

**Authors:** Aidan G O'Keeffe, Irene Petersen, Irwin Nazareth

**Affiliations:** 1 Department of Statistical Science, University College London, London, UK; 2 Department of Primary Care and Population Health, University College London, London, UK

**Keywords:** PRIMARY CARE, PREVENTIVE MEDICINE, CARDIOLOGY

## Abstract

**Objectives:**

To investigate the extent to which variation exists in the initiation rate of statin therapy for the primary prevention of cardiovascular disease between countries of the UK and between different regions within England.

**Design:**

Cohort study using data from a large UK primary care database.

**Setting:**

UK.

**Participants:**

4 820 885 individuals from 554 general practices during the period 2004–2012.

**Main outcome measures:**

Rate of statin therapy initiation per 1000 person-years.

**Results:**

Relative to a fixed English rate of 1 initiation per 1000 person-years and accounting for gender, age and social deprivation level, the rate was similar for Scotland at 0.92 (95% CI 0.84 to 1.00) and rates for Northern Ireland and Wales were higher at 1.40 (95% CI 1.20 to 1.62) and 1.18 (95% CI 1.05 to 1.32), respectively. Within England, the regions could be classified into three groups with respect to statin therapy initiation rates (relative to a rate of 1 initiation per 1000 person-years for London): the South Central 0.73 (95% CI 0.64 to 0.83), South West 0.80 (95% CI 0.71 to 0.91), East of England 0.81 (95% CI 0.71 to 0.94) and South East Coast 0.83 (95% CI 0.73 to 0.95); strategic health authorities had similar low rates followed by the East Midlands 0.88 (95% CI 0.73 to 1.05), West Midlands 0.96 (95% CI 0.84 to 1.09), North East 0.96 (95% CI 0.79 to 1.16), Yorkshire and Humber 0.97 (95% CI 0.81 to 1.17) and London strategic health authorities. North West England exhibited the highest rate of statin therapy initiation of 1.16 (95% CI 1.02 to 1.31).

**Conclusions:**

Considerable variation in the rate of statin therapy initiation was observed between the four countries of the UK and between different geographical regions within England.

**Strengths and limitations of this study**

- We conducted a large-scale analysis of statin therapy initiation rates within the UK using recent data from electronic health records. The large sample size reflects the UK general population.
- We estimated initiation rates between the different countries of the UK and between regions within England, adjusting for patient demographic variables.
- Results showed a wide variation in statin therapy initiation rates between different general practitioner (GP) practices and some differences between initiation rates in different geographical areas/countries.
- We only used data from those who are registered with a GP. However, in the UK, the vast majority of the population is registered with a GP.

## Introduction

Statins are a class of lipid-modifying drugs, commonly prescribed in UK primary care.^1^ Statins were first developed during the 1970s^2^ ^3^ and the first commercially marketed statin, simvastatin, was introduced in the UK in the late 1980s.^4^ Since then, and following results from several clinical trials,5–8 statins have become one of the most routinely prescribed class of drugs in the UK.^9^ The National Institute for Health and Care Excellence (NICE) issues guidelines on the prescription of statins for UK general practitioners (GP). In January 2006, NICE recommended that statin therapy should be initiated for the primary prevention of cardiovascular disease (CVD) in adults aged under 75 years whose 10-year risk of developing CVD, as calculated using an appropriate risk score (eg, the Framingham risk score or QRISK score), exceeds 20%.^10^ In addition, NICE recommended that individuals aged over 75 years could be considered at risk of CVD regardless of their 10-year CVD risk score and that statin therapy should be considered for this age group, particularly for individuals at risk such as smokers or hypertensives. Recently, NICE has changed these guidelines and suggested that a 10% CVD risk rule be implemented for the prescription of statins in the under 75s.

Despite the increasing public awareness of the clinical benefits of statins within the UK, there have been relatively few attempts to assess the level of initiation of statin therapy for the primary prevention of CVD within the UK population. Some studies have examined trends in statin prescription within UK general practice,11–13 but these were performed using relatively small data sets from the 1990s and early 2000s. Statin prescription trends for the secondary prevention of CVD have also been examined using small observational studies.14–16 Other studies17–19 have examined trends in the prescription of statins within Europe, without a specific focus on prescription practice within the UK. Recently, there have been some small observational studies concerning statin prescriptions within the UK,^20^ ^21^ but a recent large-scale analysis of the initiation rates of statin therapy within the UK population has not been undertaken.

Electronic health records in UK primary care allow a more thorough examination of statin use. We use such data to perform an analysis of statin therapy initiation rates for the primary prevention of CVD within the UK population from 2004 to 2012. Our principal aim is to examine whether there is any variation in the rate of initiation of statin therapy between the four UK countries and the extent to which any differences observed can be explained by demographic and/or socioeconomic factors. Furthermore, within England, we examine the extent to which statin therapy initiation rates vary between different regions.

## Data and methods

### THIN database

We used data from The Health Improvement Network (THIN) database (http://www.thin-uk.com), one of the largest sources of UK primary care data. The database contains anonymised patient records collected at over 500 UK general practices, including patient demographics, consultation information and patient therapy records, linked over time. Over 98% of the UK population are registered with a general practice^22^ and THIN is regarded as broadly representative of the general UK population.^23^ The database also includes information on the Townsend score, a measure of social deprivation, categorised into five quintiles from one (least deprived) to five (most deprived).

### Our study

We chose to examine statin initiation levels in individuals with no history of CVD events during the period 2004–2012. We chose to begin our analyses in 2004 because of the introduction of the Quality and Outcomes Framework (QOF) at that time. Patients who initiated statins within 6 months of their date of registration at a practice were excluded. This was done in an effort to ensure that patients who moved practice and who had previously initiated statin therapy would not be included. The use of a 6-month ‘qualification period’ appears reasonable, since the vast majority of individuals receive statins on monthly or at most three monthly prescriptions and a 6-month ‘qualification period’ has been used in other studies that used THIN.24–26 The principal outcome of interest is the number of statin therapy initiations during the time period 2004–2012. Time is accounted for explicitly by measuring the total patient time ‘at risk’ (where ‘at risk’ implies ‘eligible for statin therapy initiation’) during this period and the time until first statin prescription or end of follow-up at the patient level. Important patient level and practice level variables such as gender, age, Townsend score quintile, UK country and strategic health authority (SHA; English practices) were also recorded. We note that SHAs were abolished in March 2013; however, we feel that SHAs remain a useful variable for a comparison of English regions.

### Statistical analyses

The main outcome of interest is the statin therapy *initiation rate* per 1000 person-years. For each individual, the person-years of follow-up were calculated as running *from* the latest of: 18th birthday, practice registration date or 1 January 2004 *to* the earliest of: date of first statin prescription, date of death, date of first CVD event, date of transfer from practice or 31 December 2012. As such, only those adult patients who had neither experienced a CVD event nor had previously been prescribed statins were included. The statin therapy initiation rate was estimated both within each practice and using data from all practices. Furthermore, statin therapy initiation rates were estimated within each country of the UK and for each strategic health authority within England.

We used Poisson regression models, with log (person-years at risk) as an offset term, to estimate the rate of statin therapy initiation with regard to gender, age group and Townsend score quintile. To account for clustering of individuals within practices, we included practice level random effects. Age was classified into 12 groups according to age in years (18–39, 40–49, 50–54, 55–59, 60–64, 65–69, 70–75, 75–80, 80–85, 85–90, 90–95 and 95+).

English practices contributed the majority of data and so we felt that it was appropriate to examine possible differences in statin therapy initiation rates between different regions of England. Before 1 April 2013, primary care within England was governed by 10 SHAs: London, East Midlands, West Midlands, East of England, North East England, North West England, South Central, South East Coast, South West England and Yorkshire and Humber. Although SHAs have since been replaced by smaller clinical commissioning groups, the SHAs existed while our data were collected and we feel that a comparison of rates between different SHAs could be important in highlighting possible differences in statin therapy initiation rates within England. Table 1 summarises the number of practices within the different SHAs in our data.

**Table 1:** The number of practices within each strategic health authority (SHA)

SHA	Number of practices (percentage of English practices)
London	63 (15.51)
East Midlands	19 (4.68)
East of England	36 (8.87)
West Midlands	44 (10.84)
North East	15 (3.69)
North West	61 (15.02)
Yorkshire and Humber	17 (4.19)
South Central	51 (12.56)
South East Coast	47 (11.58)
South West	53 (13.05)

Data manipulation was carried out using STATA V.13 with other statistical analyses, including all modelling, undertaken using the R statistical package (V.3.0.2).

## Results

We analysed data from 554 GP practices across the UK (by country: England: 406, Northern Ireland: 23, Scotland: 85, Wales: 40) using records from 4 820 885 individuals. Table 2 provides a summary of cohort demographics stratified by UK country. There were slightly more females than males included in our analyses, possibly because, in general, females are more likely than males to consult their GP regularly.^27^ Age at cohort entry was broadly similar between countries. We see that, across all countries, there were similar proportions of patients in Townsend score quintiles 2–4 with slightly larger and smaller proportions in quintiles 1 and 5, respectively. This distribution of Townsend score quintiles is seen within England but not for the other countries of the UK, with Northern Ireland, Scotland and Wales tending to have higher proportions of patients in more deprived Townsend score quintiles.

**Table 2:** Demographics

Variable	UK country				
	All	England	Northern Ireland	Scotland	Wales
Gender counts					
Male	2 312 378 (47.97%)	1 826 391 (47.89%)	55 350 (47.69%)	275 158 (48.59%)	155 479 (47.87%)
Female	2 508 507 (52.03%)	1 987 390 (52.11%)	60 724 (52.31%)	291 089 (51.41%)	169 304 (52.13%)
Mean age at cohort entry (years)	40.2	40.4	39.0	40.1	38.7
Townsend quintile counts					
1 (least deprived)	1 121 219 (23.26%)	966 693 (25.35%)	25 402 (21.88%)	68 734 (12.14%)	60 390 (18.59%)
2	993 426 (20.61%)	776 252 (20.35%)	21 946 (18.91%)	129 204 (22.82%)	66 024 (20.33%)
3	1 024 050 (21.24%)	800 858 (21.00%)	24 364 (20.99%)	119 206 (21.05%)	79 622 (24.52%)
4	980 006 (20.33%)	756 345 (19.83%)	18 535 (15.97%)	125 519 (22.17%)	79 607 (24.51%)
5 (most deprived)	702 184 (14.57%)	513 633 (13.47%)	25 827 (22.25%)	123 584 (21.83%)	39 140 (12.05%)
Total number of patients	4 820 885				

**Figure 1:**
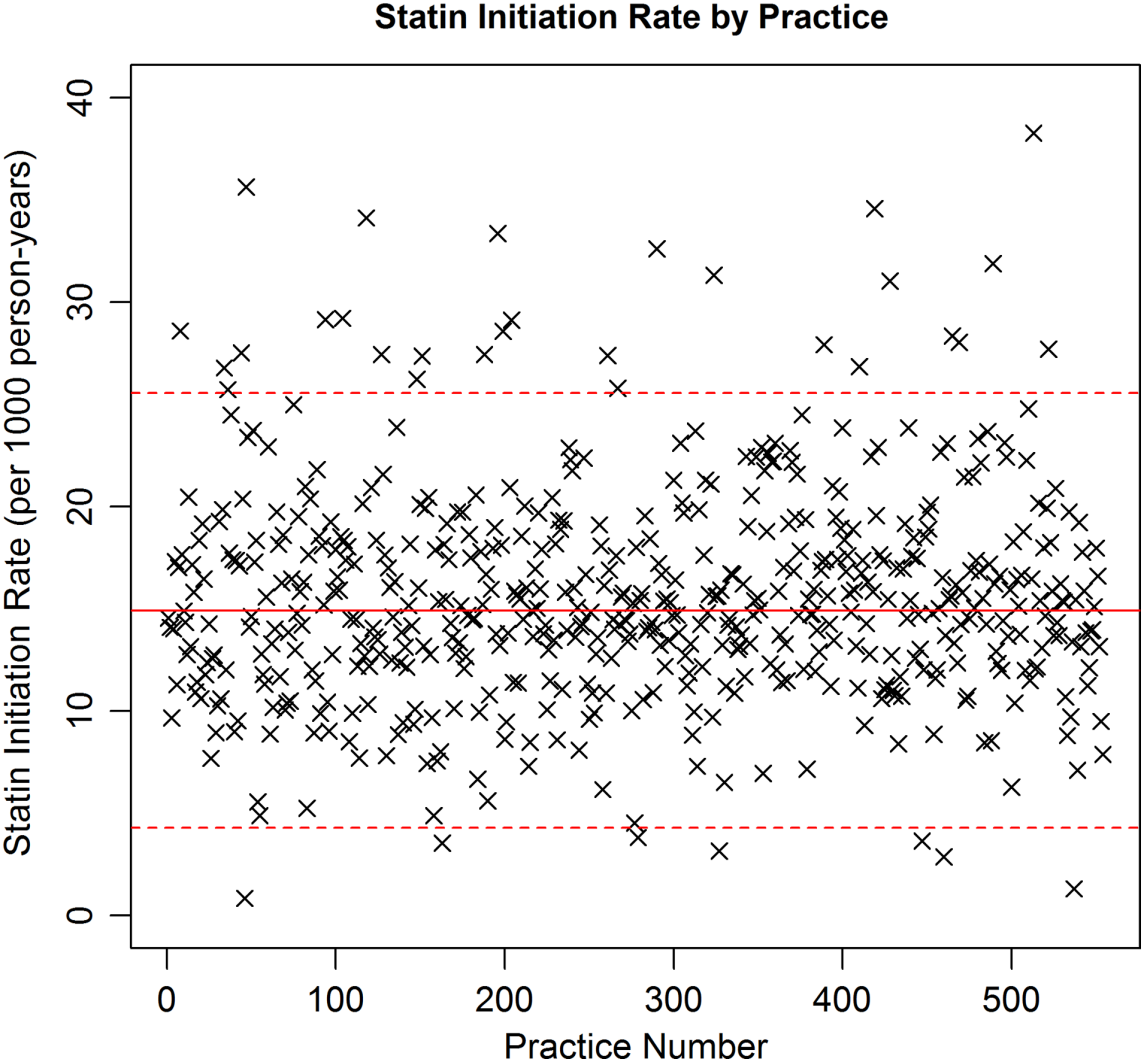
Plot showing the estimated statin therapy initiation rate for each of the 554 general practitioner practices. The solid red line indicates the sample mean statin therapy initiation rate. The dashed red lines indicate this sample mean±2SD(sample SD).

**Figure 2:**
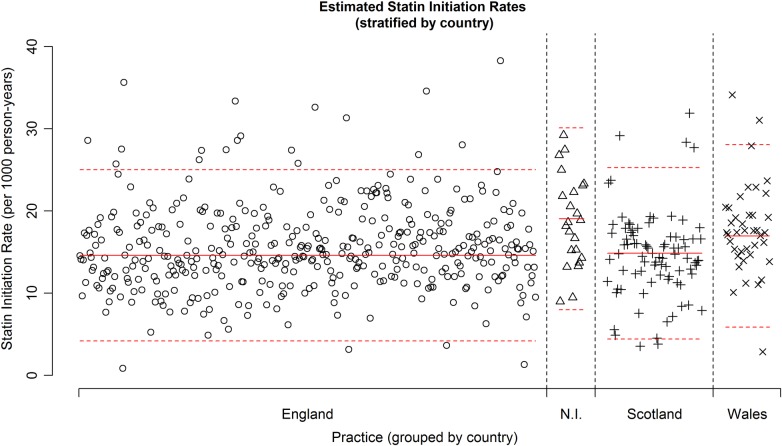
Plot showing the estimated statin therapy initiation rate for each of the 554 general practitioner practices, stratified by UK country. The solid red line indicates the sample mean statin therapy initiation rate within a country. The dashed red lines indicate this sample mean ±2SD(sample SD) within a country.

Figure 1 shows a plot of the estimated statin therapy initiation rate by practice, with a solid red line showing the mean estimated statin therapy initiation rate using data from all practices and the dashed red lines showing this mean ±2SD(sample SD). Although many practices have estimated initiation rates close to the overall mean rate of 14.91 per 1000 person-years, there exists substantial variation in rates between different practices, with estimated initiation rates ranging from 0.83 to 38.26 initiations per 1000 patient-years. Seven practices have a rate lower than two sample SDs below the estimated mean initiation rate and 28 practices have a rate greater than two sample SDs above the estimated mean initiation rate. Figure 2 shows a similar plot, but with practices grouped according to UK country. Practices within England and Scotland yield very similar mean estimated initiation rates, whereas those in Northern Ireland and Wales have a slightly higher mean estimated initiation rate. In addition, variation in estimated rates appears slightly smaller within Northern Ireland, although we note the smaller number of Northern Irish practices.

Table 3 shows results from fitted models. A comparison of the adjusted and unadjusted initiation rates shows that even after accounting for patient demographics, initiation rates within Northern Ireland and Wales are greater than that in England, whereas the rate in Scotland is slightly lower than that in England.

**Table 3:** Summary of country results

Country	Estimated statin initiation rate (estimated 95% CI)	Unadjusted relative statin initiation rates	Adjusted relative statin initiation rates*
England	14.82 (14.37 to 15.28)	1	1
Northern Ireland	18.52 (16.23 to 21.15)	1.25 (1.09 to 1.43)	1.40 (1.20 to 1.62)
Scotland	14.63 (13.64 to 15.69)	0.99 (0.91 to 1.07)	0.92 (0.84 to 1.00)
Wales	17.06 (15.49 to 18.78)	1.15 (1.04 to 1.27)	1.18 (1.05 to 1.32)

The second column shows unadjusted estimated rates for each country, with the third column showing rates for Scotland, Northern Ireland and Wales relative to a fixed rate of 1 unit per 1000 person-years for England. The fourth column shows these relative rates but where rates are adjusted to account for gender, age group in years and Townsend score quintile.

*Rates adjusted for gender, age group and Townsend score quintile.

### Regions within England

**Figure 3:**
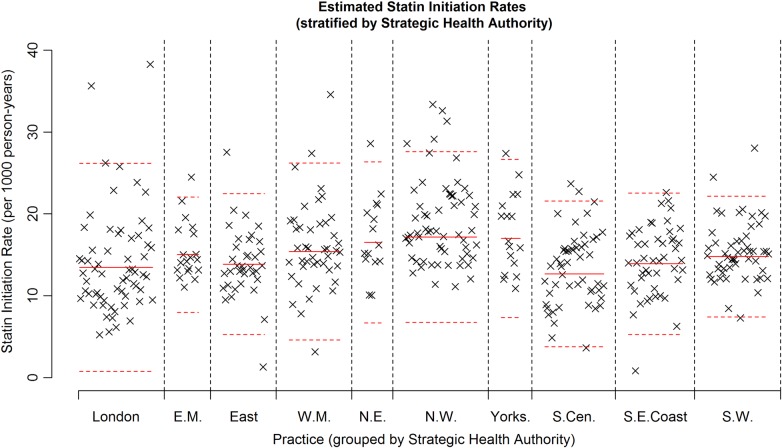
Plot showing the statin initiation rate for each practice, stratified by strategic health authority (indicated by an icon for each practice). The solid red lines show the estimate of the statin initiation rate using data from within a strategic health authority. The dashed red lines indicate sample mean ±2SD(sample SD) within a strategic health authority (EM, East Midlands; NE, North East; NW, North West; SCen, South Central; SECoast, South East Coast; SW, South West; WM, West Midlands).

Figure 3 shows a plot of the estimated statin therapy initiation rate by practice within England, but with practices grouped according to SHA. Solid red horizontal lines denote the within SHA mean initiation rate and red dashed lines denote a mean ±2SD(sample SD) for initiation rate within each SHA. This plot shows that there is considerable variation in initiation rates between practices within each region. Table 4 provides a numerical comparison of estimated initiation rates, with a practice-specific random effect to account for the clustering of patients within practices. Prior to adjustment for the demographic variables (columns 2 and 3), we see that observable differences in initiation rates exist between the different SHAs, with the West Midlands, Yorkshire and Humber, North West and North East SHAs showing slightly higher rates than those seen in London and the South of England. After accounting for demographic variables (column 4), these differences are less marked, although the North West region appears to have a significantly higher initiation rate when compared to London. Furthermore, the East of England, South Central, South East Coast and South West SHAs exhibit significantly lower initiation rates when compared to the London, East Midlands, West Midlands, Yorkshire and Humber and North East SHAs, even after accounting for demographic variables.

**Table 4:** Estimated statin therapy initiation rates for different strategic health authorities (SHA) within England

Strategic health authority	Statin initiation rate (estimated 95% CI)	Unadjusted relative statin initiation rate	Adjusted relative statin initiation rate*
London	13.33 (12.31 to 14.43)	1	1
East Midlands	15.17 (13.30 to 17.30)	1.14 (0.98 to 1.33)	0.88 (0.73 to 1.05)
East of England	13.91 (12.58 to 15.37)	1.04 (0.92 to 1.19)	0.81 (0.71 to 0.94)
West Midlands	15.76 (14.44 to 17.21)	1.18 (1.05 to 1.33)	0.96 (0.84 to 1.09)
North East	16.47 (14.17 to 19.15)	1.24 (1.04 to 1.47)	0.96 (0.79 to 1.16)
North West	18.03 (16.72 to 19.44)	1.35 (1.21 to 1.51)	1.16 (1.02 to 1.31)
Yorkshire and Humber	17.37 (15.07 to 20.01)	1.30 (1.11 to 1.53)	0.97 (0.81 to 1.17)
South Central	12.75 (11.77 to 13.81)	0.96 (0.86 to 1.07)	0.73 (0.64 to 0.83)
South East Coast	13.78 (12.66 to 15.00)	1.03 (0.92 to 1.16)	0.83 (0.73 to 0.95)
South West	14.99 (13.83 to 16.25)	1.13 (1.00 to 1.26)	0.80 (0.71 to 0.91)

The first column shows overall rate estimates, the second column shows relative statin therapy initiation rates and the third column shows statin therapy initiation rates adjusted for chosen factors. The second column shows unadjusted estimated rates for each SHA, with the third column showing rates for non-London SHAs relative to a fixed rate of 1 unit per 1000 person-years for the London SHA. The fourth column shows these relative rates but where rates are adjusted to account for gender, age group and Townsend score quintile.

*Rates adjusted for gender, age group and Townsend score quintile.

### Gender, age group and Townsend score quintile

Finally, we examined the statin therapy initiation rates controlling for gender, age group and the Townsend score quintile, including a practice level random effect. Table 5 provides a summary of these results. For age group, we see that the initiation rate generally increases with age, and this occurs until patients reach 75 years whence rates begin to decrease. Males tend to have higher estimated initiation rates than females. Initiation rates for males appear to decrease slightly as the Townsend score quintile increases (ie, as areas become more socially deprived); however, once age distribution is accounted for (5th main column of table 5), this trend is no longer seen. Estimated unadjusted statin therapy initiation rates are broadly similar for females across all Townsend score quintiles. However, age-adjusted statin therapy initiation rates appear to increase in females as the Townsend score quintile increases (ie, as areas become more socially deprived).

**Table 5:** Estimated statin therapy initiation rates for age groups and Townsend score quintiles, stratified according to gender

Age group (years)	Statin initiation rate per 1000 person-years (estimated 95% CI)		Unadjusted relative statin initiation rate		Adjusted relative statin initiation rate*	
	Males	Females	Males	Females	Males	Females
18–39	1.63 (1.56 to 1.70)	0.93 (0.89 to 0.98)	1	0.57 (0.55 to 0.60)	1	0.57 (0.55 to 0.60)
40–49	10.79 (10.42 to 11.17)	6.41 (6.18 to 6.65)	6.63 (6.43 to 6.83)	3.94 (3.81 to 4.07)	6.69 (6.49 to 6.89)	3.98 (3.85 to 4.11)
50–54	23.03 (22.23 to 23.86)	15.87 (15.29 to 16.46)	14.15 (13.71 to 14.60)	9.75 (9.43 to 10.08)	14.31 (13.88 to 14.77)	9.88 (9.56 to 10.21)
55–59	34.69 (33.51 to 35.91)	25.36 (24.47 to 26.27)	21.32 (20.68 to 21.98)	15.58 (15.10 to 16.08)	21.60 (20.95 to 22.26)	15.82 (15.33 to 16.32)
60–64	51.63 (49.89 to 53.42)	36.33 (35.08 to 37.61)	31.72 (30.78 to 32.69)	22.32 (21.64 to 23.02)	32.17 (31.23 to 33.14)	22.68 (22.00 to 23.38)
65–69	67.11 (64.82 to 69.47)	48.26 (46.60 to 49.98)	41.24 (39.99 to 42.52)	29.65 (28.75 to 30.59)	41.80 (40.55 to 43.08)	30.10 (29.19 to 31.03)
70–74	70.77 (68.28 to 73.35)	57.43 (55.44 to 59.50)	43.48 (42.12 to 44.89)	35.29 (34.20 to 36.42)	44.04 (42.68 to 45.46)	35.70 (34.61 to 36.83)
75–79	43.64 (41.91 to 45.44)	47.69 (45.95 to 49.51)	26.81 (25.84 to 27.83)	29.31 (28.34 to 30.31)	27.10 (26.12 to 28.11)	29.52 (28.56 to 30.52)
80–84	32.39 (30.89 to 33.97)	34.83 (33.43 to 36.28)	19.90 (19.04 to 20.81)	21.40 (20.61 to 22.22)	20.06 (19.20 to 20.96)	21.45 (20.67 to 22.26)
85–89	22.10 (20.65 to 23.65)	23.54 (22.39 to 24.75)	13.58 (12.71 to 14.50)	14.47 (13.80 to 15.17)	13.66 (12.80 to 14.57)	14.46 (13.80 to 15.16)
90–94	12.47 (10.85 to 14.32)	11.20 (10.26 to 12.24)	7.66 (6.67 to 8.79)	6.88 (6.31 to 7.51)	7.69 (6.71 to 8.81)	6.88 (6.31 to 7.49)
95+	4.51 (2.89 to 7.04)	5.03 (4.08 to 6.21)	2.77 (1.78 to 4.32)	3.09 (2.51 to 3.82)	2.77 (1.79 to 4.30)	3.09 (2.51 to 3.81)

Townsend score quintile	Statin initiation rate per 1000 person-years (estimated 95% CI)		Unadjusted relative statin initiation rate		Adjusted relative statin initiation rate†	
	Males	Females	Males	Females	Males	Females
1	18.15 (17.45 to 18.87)	13.26 (12.72 to 13.82)	1	0.73 (0.70 to 0.76)	1	0.66 (0.65 to 0.67)
2	17.52 (16.82 to 18.24)	13.76 (13.19 to 14.36)	0.97 (0.92 to 1.01)	0.76 (0.72 to 0.79)	1.01 (0.99 to 1.02)	0.70 (0.69 to 0.72)
3	16.01 (15.35 to 16.70)	13.69 (13.11 to 14.29)	0.88 (0.84 to 0.92)	0.75 (0.72 to 0.79)	1.02 (1.01 to 1.04)	0.78 (0.76 to 0.79)
4	15.19 (14.52 to 15.88)	14.03 (13.41 to 14.68)	0.84 (0.80 to 0.88)	0.77 (0.74 to 0.81)	1.05 (1.03 to 1.07)	0.86 (0.85 to 0.88)
5	14.68 (13.93 to 15.46)	14.40 (13.66 to 15.18)	0.81 (0.76 to 0.86)	0.79 (0.75 to 0.84)	1.07 (1.05 to 1.09)	0.94 (0.92 to 0.96)

The first column shows overall rate estimates, the second column shows relative statin therapy initiation rates and the third column shows statin therapy initiation rates adjusted for chosen factors.

*Rates adjusted for Townsend score quintile.

†Rates adjusted for age group.

## Discussion

### Findings

We found considerable variation in statin therapy initiation rates between different practices across the UK. These rates were similar in England and Scotland but in Wales and Northern Ireland were 18% (95% CI 5% to 32%) to 40% (95% CI 20% to 62%) higher, even after accounting for the possible differences in patient demographics and the clustering of patients with practices, between countries. Considerable variation in the initiation rate was observed between different English regions after accounting for differences in patient demographics. The North West of England had a significantly higher initiation rate when compared to London, Yorkshire and Humber, North East England, East Midlands and West Midlands SHAs. Furthermore, the East of England, South Central, South East Coast and South West SHAs had significantly lower estimated initiation rates when compared to this group of SHAs.

Summary statistics have indicated that regional differences in CVD morbidity exist within the UK.^28^ It is possible that some difference in statin therapy initiation rates may be explained by the difference in underlying CVD risk within the population served by the practices, such as dietary habits, the levels of smoking and diabetes, low-density lipoprotein (LDL) cholesterol level, hypertension and body mass index (BMI) among others. Previous research has shown that differences between practice prescribing rates for statins were positively related to the rate of coronary heart disease (CHD) hospital diagnoses and negatively associated with deprivation.^13^ ^29^ ^30^ Examining data from 2010, Scotland has a higher rate of myocardial infarction than England, and similarly the North East and North West regions of England have higher rates of myocardial infarction with the South East and East of England reporting the lowest rates.^28^ Although our study focused on statin prescription for primary prevention, our findings concur with some of these observations.

Initiation rates were generally lower in females compared to males and rates tended to increase with age for both genders, until approximately 75 years, after which rates generally declined. After accounting for age group, initiation rates increased slightly as the Townsend score quintile increased for both males and females. The risk of CHD among middle-aged people is 2–5 times more common in males than females, but for both genders the risk of CHD increases with age and the risk of CHD in older males and females is similar.^31^ Our findings suggest that prescribing was lower in women than men across all age groups with the exception of those over 75 years of age who were prescribed statins at the same rate irrespective of their gender. The lower rates of prescribing in the over 75s, despite their heightened risk of CHD, might suggest that primary prevention of CVD in the elderly could be improved. Nevertheless, our findings represent an improvement in practice. A previous recent study suggested that patients over 75 years of age were unlikely to be prescribed a statin for the primary prevention of CVD irrespective of their gender.^13^ It is likely that, with the absence of risk assessment methods in older people, GP could benefit from clearer guidance on risk assessment for people above the age of 75 years.

Our data clearly indicate that initiation of stain therapy increased with deprivation (after accounting for age). Once again, this suggests a change in GPs’ prescribing behaviour. Previous data suggested that practices with higher deprivation indices prescribed fewer statins to their patients than those in less deprived practices.^32^ However, our findings suggest that these inequalities in prescribing based on deprivation have narrowed since the study by Packham *et al.*

### Strengths and limitations

A clear strength of this study is the large sample size which reflects the general UK population and allows the modelling of quantities of interest, controlled for a range of factor variables, without the problem of small group sizes in the model fitting process. In addition, since most GPs generate prescriptions electronically, the nature of the THIN database allows us to accurately monitor statin prescriptions in a detailed manner, by patient, longitudinally. We feel that this has allowed us to make informed and comprehensive assessment statin therapy initiation rates for primary prevention of CVD, within the UK population.

There are limitations to our methods and sample. We are only using data from those who are registered with a GP. However, in the UK, the vast majority of the population is registered with a GP. We have accounted for sociodemographic variables such as age, sex and social deprivation. While these may be strongly correlated to other factors that may determine initiation of statin prescriptions, it is possible that primary initiation of statins may be governed by factors that we have not captured by the sociodemographic variables.

It is possible that the rate of statin therapy initiation is related to measures such as smoking status, BMI, blood pressure and LDL cholesterol level among others. While it may have been desirable, at first sight, to include such variables in our analyses, we bear in mind the limitations of primary care records. In particular, measures such as BMI, blood pressure and LDL cholesterol level are not recorded for all individuals and may be more likely to exist for those patients who visit their GP more regularly than others, or perhaps those who suffer from chronic or recurrent health conditions.^33^ ^34^ Naturally, such patients would not be representative of the general UK population and, as such, the inclusion of variables such as these in our analyses might lead to substantial biases. Conversely, basic demographic variables, such as age group, gender and social deprivation status (taken from a patient's postcode), are available for the vast majority of individuals who are registered with a GP, irrespective of any health conditions exhibited by a patient, thereby justifying the use of such variables in our analyses.

### Final conclusions

There is a considerable variation in the rate of initiation of statins for primary prevention of CVD in 4 UK countries and the 10 English regions. However, at the individual level, there is insufficient evidence to suggest that inequalities in prescribing exist between the sexes and across various levels of deprivation. GP may benefit from guidance on statin prescribing in people above the age of 75 years.
